# Supplementary material for: Parasitic structure defect blights sustainability of cobalt-free single crystalline cathodes
Source: Nat Commun. 2025 Jan 6;16:434. doi: 10.1038/s41467-024-55235-5 (PMC11704202; doi:10.1038/s41467-024-55235-5)
Supplement: Supplementary file 1 — Supplementary Information [file 41467_2024_55235_MOESM1_ESM.pdf]

# Supplementary information

## Parasitic Structure Defect Blights Sustainability of Cobalt-Free Single Crystalline Cathodes

*Lei Yu[1#], Alvin Dai[2#], Tao Zhou[1], Weiyuan Huang[2], Jing Wang[2], Tianyi Li[3], Xinyou He[4], Lu Ma[5], Xianghui Xiao[5], Mingyuan Ge[5], Rachid Amine[6], Steven N. Ehrlich[5], Xing Ou[4]*, Jianguo Wen[1]*, Tongchao Liu[2]*, Khalil Amine[2]**

[1]*Center for Nanoscale Materials, Argonne National Laboratory, Lemont, IL, 60439, USA*

[2]*Chemical Sciences and Engineering Division, Argonne National Laboratory, Lemont, IL, 60439, USA*

[3]*X-ray Science Division, Advanced Photon Sources, Argonne National Laboratory, Lemont, IL, 60439, USA*

[4]*Engineering Research Center of the Ministry of Education for Advanced Battery Materials, School of Metallurgy and Environment, Central South University, Changsha, 410083 P. R. China*

[5]*National Synchrotron Light source II, Brookhaven National Laboratory, Upton, NY 11973, USA*

[6]*Material Science Division, Argonne National Laboratory, Lemont, IL 60439, USA*

*Corresponding author: ouxing@csu.edu.cn (X. O.); jwen@anl.gov (J. W.); liut@anl.gov (T. L.); amine@anl.gov (K. A.)

**Supplementary Table 1 |** The refinement results of the HEXRD measurements of SC75, including lattice parameters, Li/Ni disorder and $R_{wp}$.

| Samples | a=b | c | Li/Ni disorder | $R_{wp}$ |
|---|---|---|---|---|
| SC75 | 2.8820 | 14.2447 | 5.44% | 4.83% |

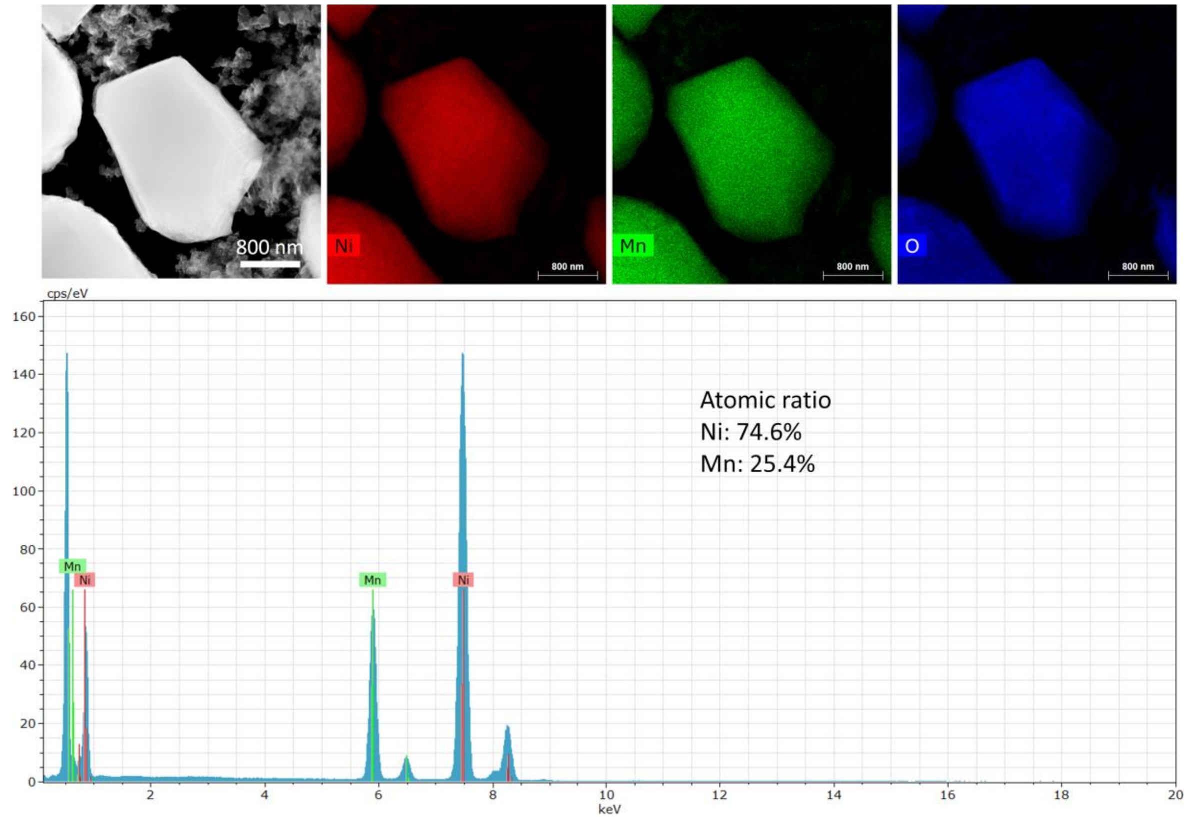


**Supplementary Fig. 1 |** EDS element analysis of the SC75 sample.

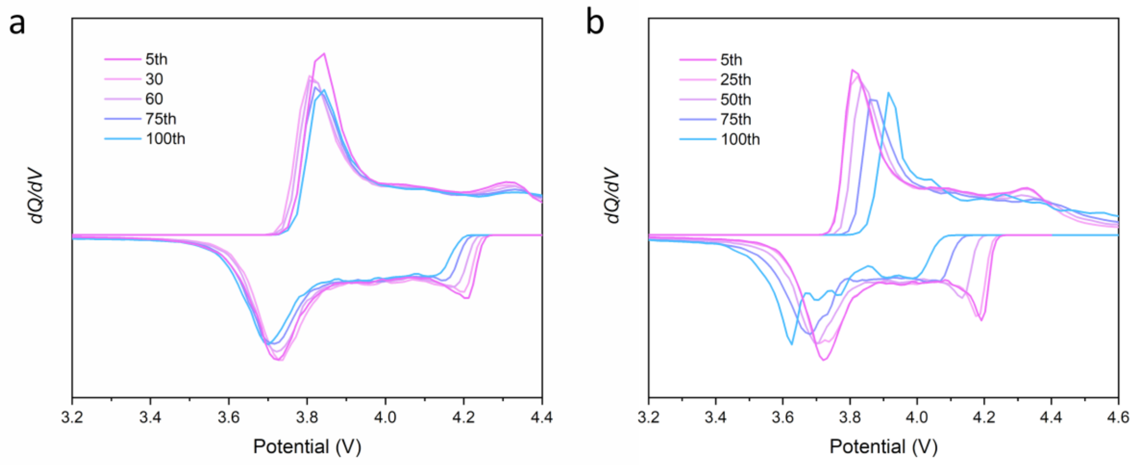


**Supplementary Fig. 2 |** The *dQ/dV* curves for 4.4 V and 4.6 V cycling, respectively.

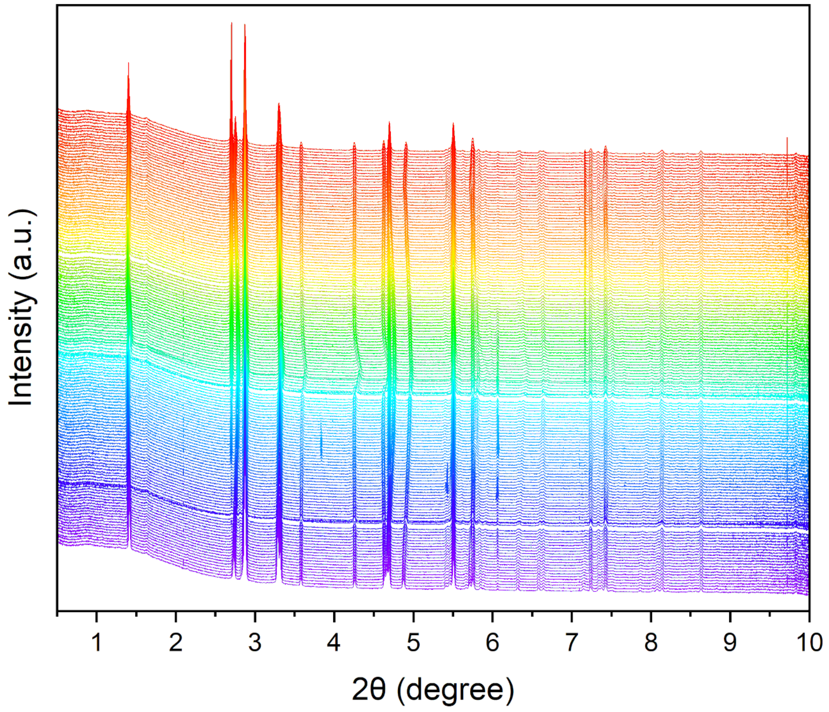


**Supplymentary Fig. 3 |** The in situ HEXRD patterns of SC75 during the first charge/discharge in the voltage range of 2.8-4.4 V using a current rate of 0.1C (1C = 200 mA $g^{-1}$). The obvious lattice parameter changes can be observed from in situ HEXRD patterns, particularly in the 2θ range of 3.0-6.0.

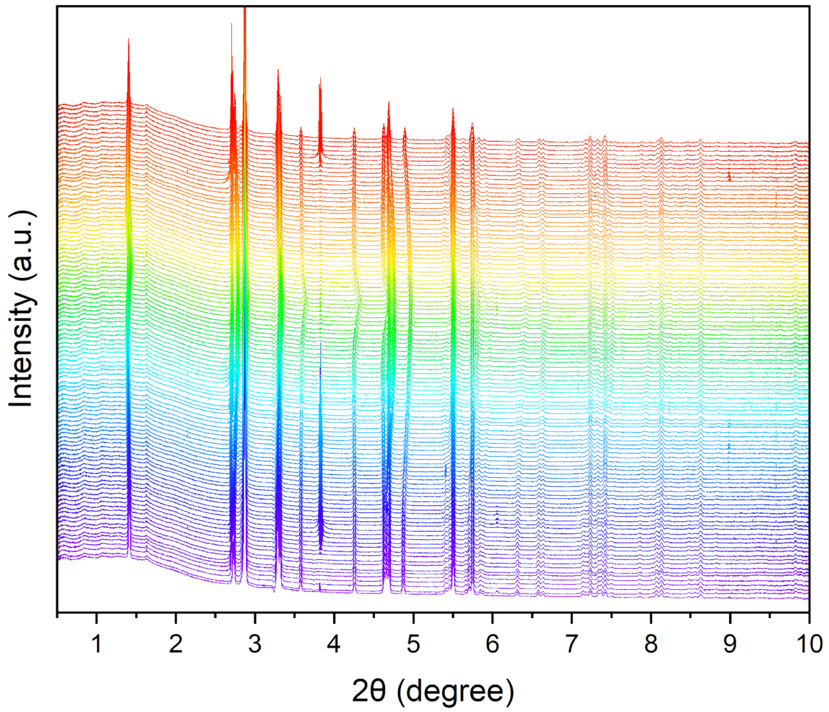


**Supplymentary Fig. 4 |** The in situ HEXRD patterns of SC75 during the first charge/discharge in the voltage range of 2.8-4.6 V using a current rate of 0.1C (1C = 200 mA $g^{-1}$). The obvious lattice parameter changes can be observed from in situ HEXRD patterns, particularly in the 2θ range of 3.0-6.0.

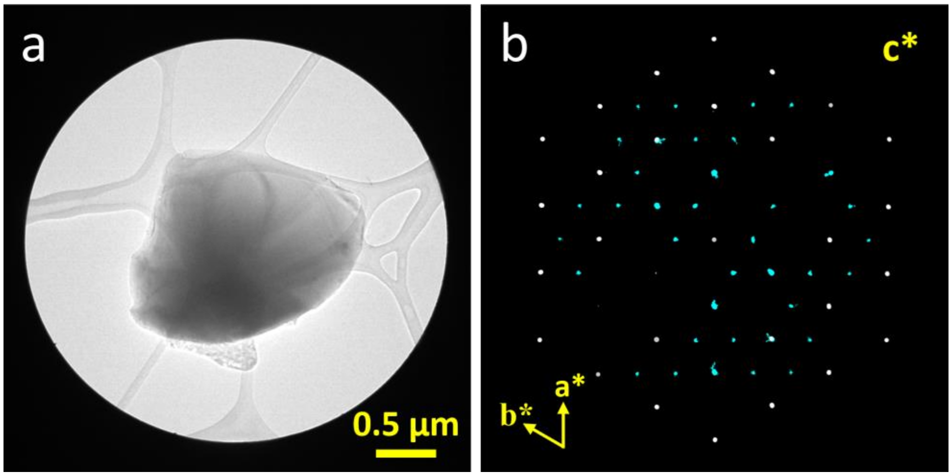


**Supplymentary Fig. 5 | a**, The TEM image of a SC75 particle for 3D-CRED. **b**, The reconstructed 3D-reciprocal lattice projection along the *c** direction.

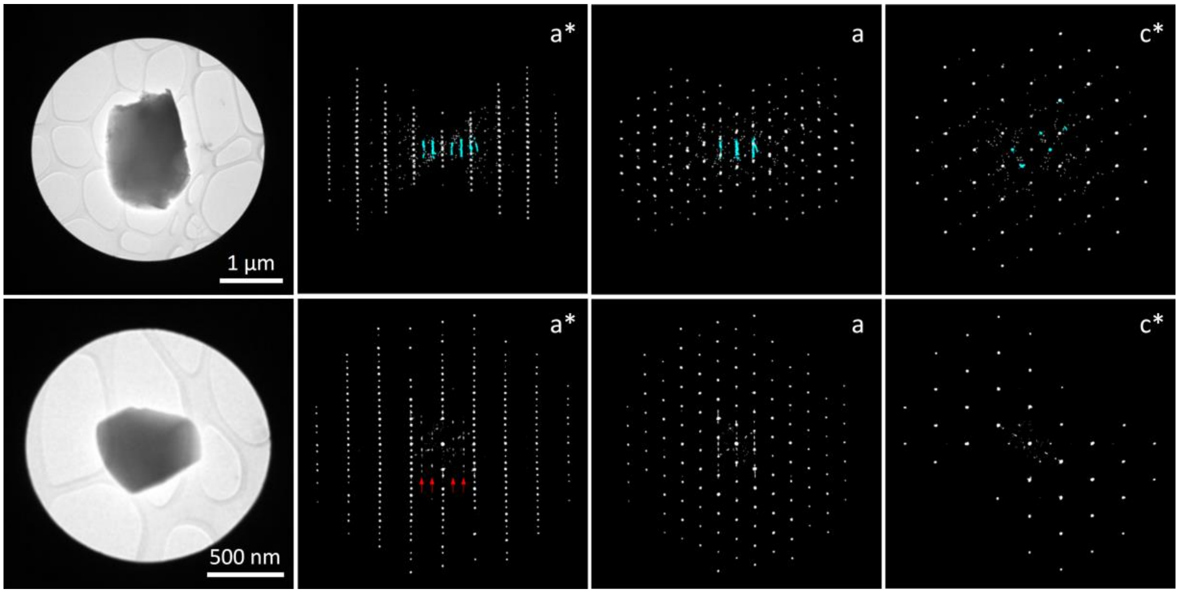


**Supplymentary Fig. 6 |** The 3D-CRED results of other randomly selected SC75 particles.

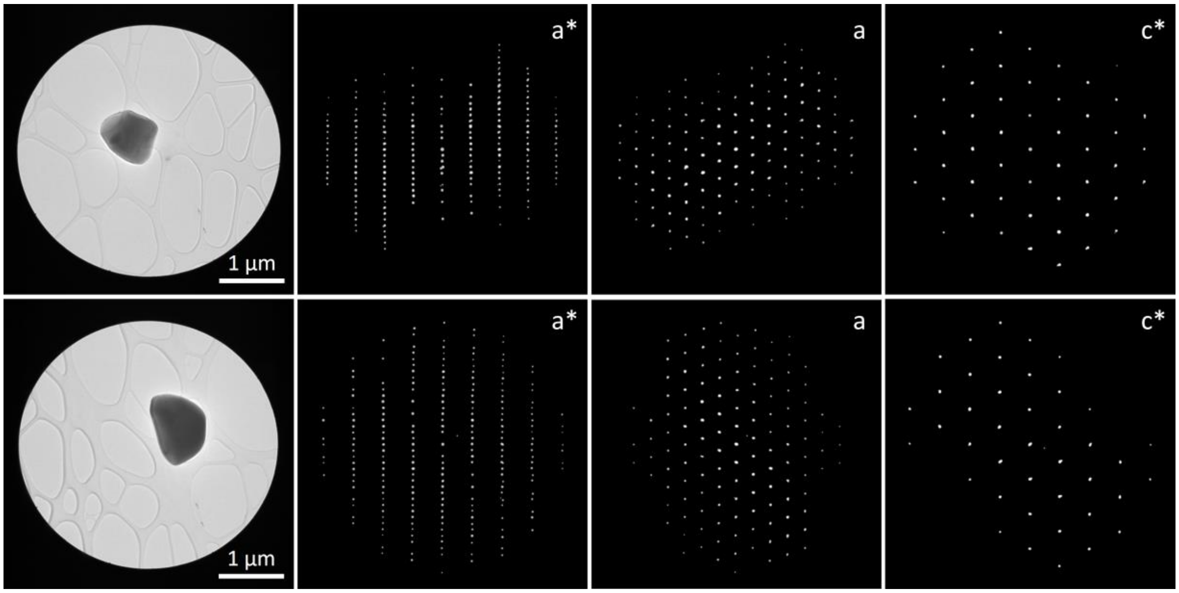


**Supplymentary Fig. 7 |** The 3D-CRED results of Co-containing single crystalline $LiNi_{0.81}Mn_{0.06}Co_{0.13}O_2$, showing typical layered $LiTMO_2$ diffraction lattice without extra structural defects.

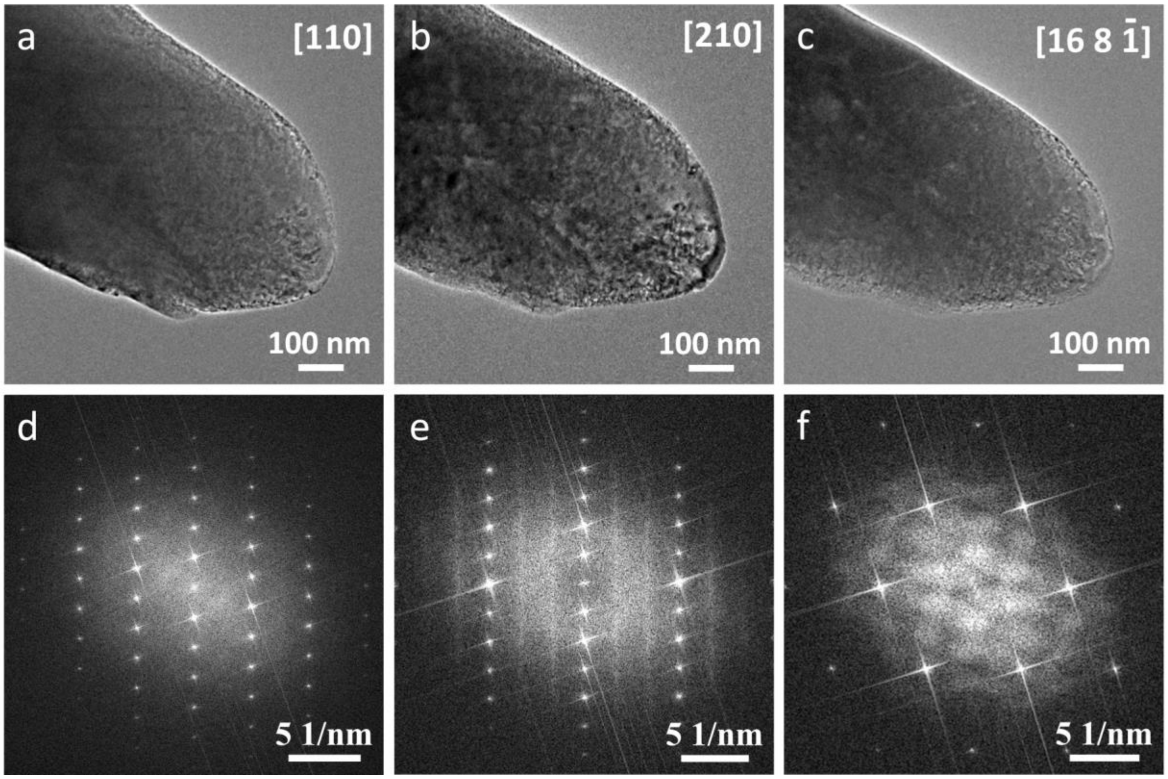


**Supplymentary Fig. 8 | a-c**, The low-magnification TEM images of a same SC75 particle along different zone axes. **d-f**, The corresponding fast fourier transformation (FFT) patterns of the HRTEM images in the **Fig. 3g-i**.

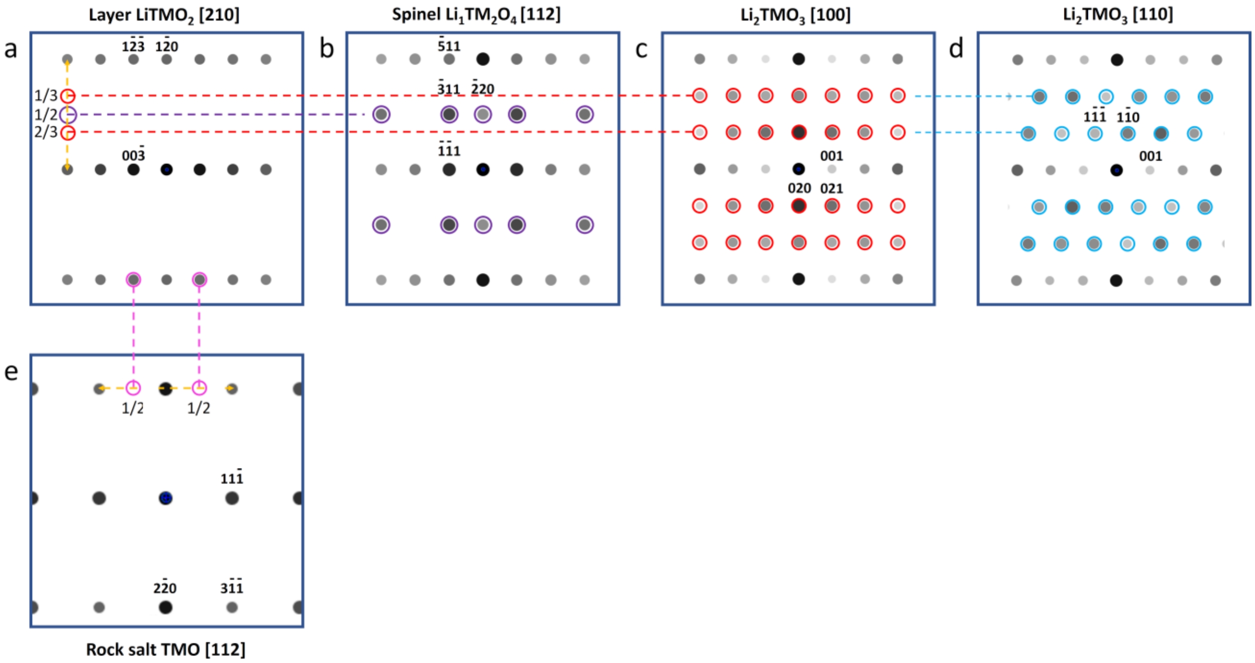


**Supplymentary Fig. 9 |** The simulated patterns of standard electron diffractions for layered $LiTMO_2$ [210], spinel $Li_1TM_2O_4$ [112], Li-rich $Li_2TMO_3$ [100]/[110] and rock salt TMO [112].

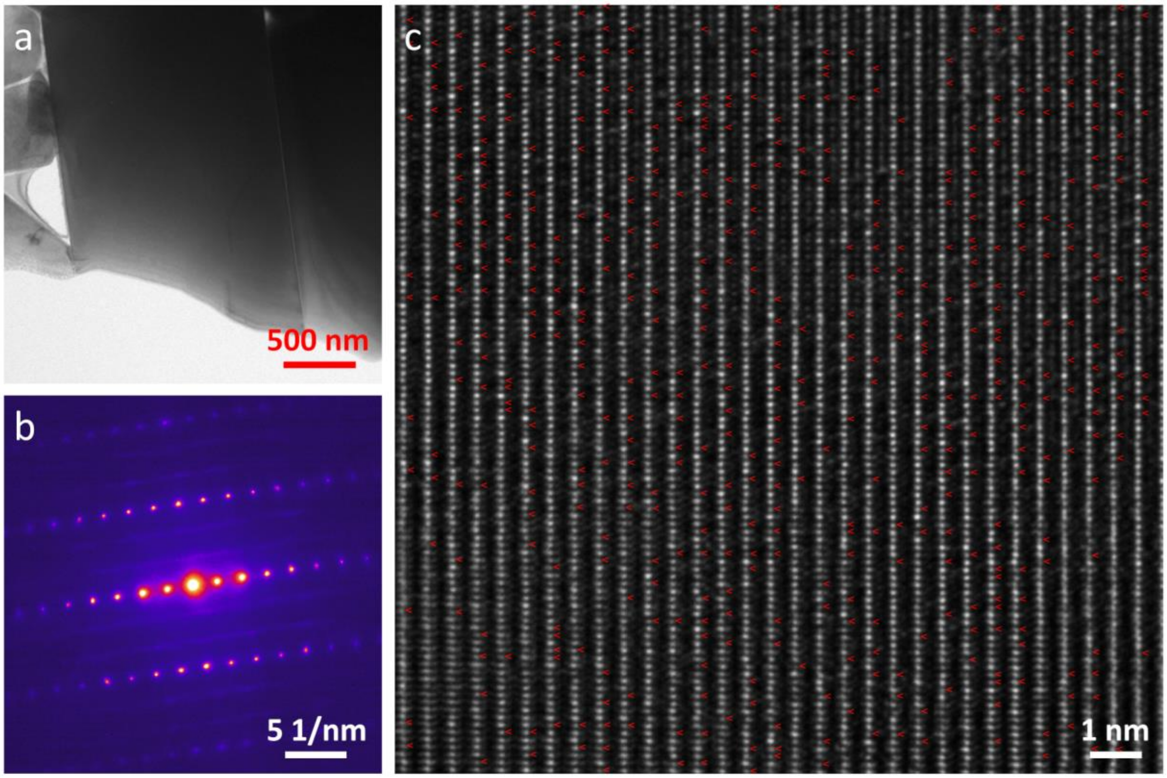


**Supplymentary Fig. 10 | a**, The low-magnification TEM image of the SC75 particle along the [210] zone axis. **b**, The corresponding SAED pattern. **c**, The HRTEM image with red indicators showing the dim dots.

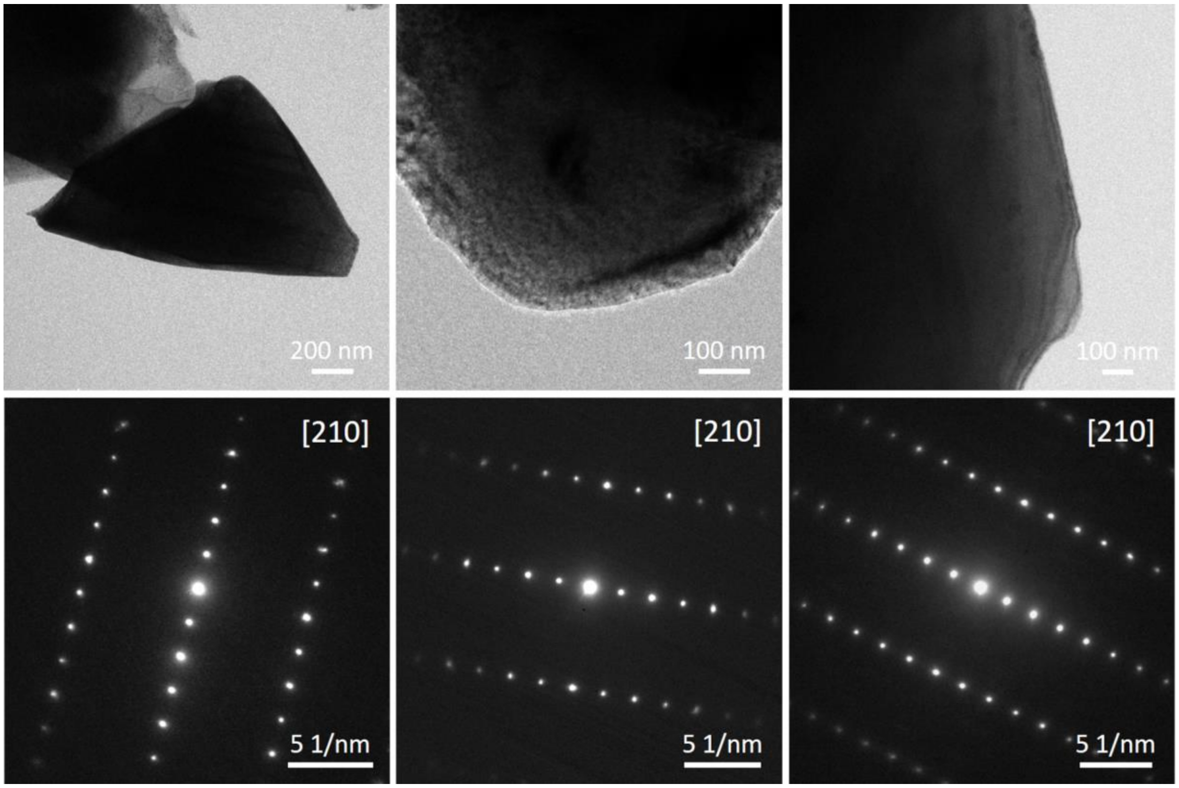


**Supplymentary Fig. 11 |** The SAED results of Co-contianing single crystalline $LiNi_{0.81}Mn_{0.06}Co_{0.13}O_2$ along the [210] zone axis, showing no obvious streak diffraction.

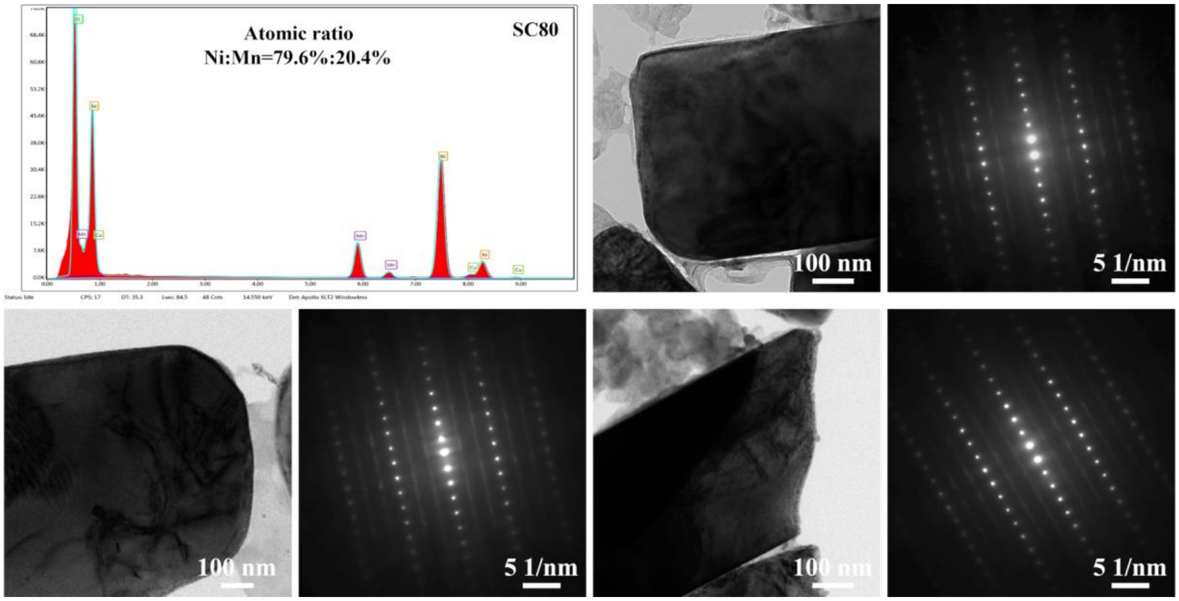


**Supplementary Fig. 12 |**The EDS spectrum and structural characterization of the SC80 sample.

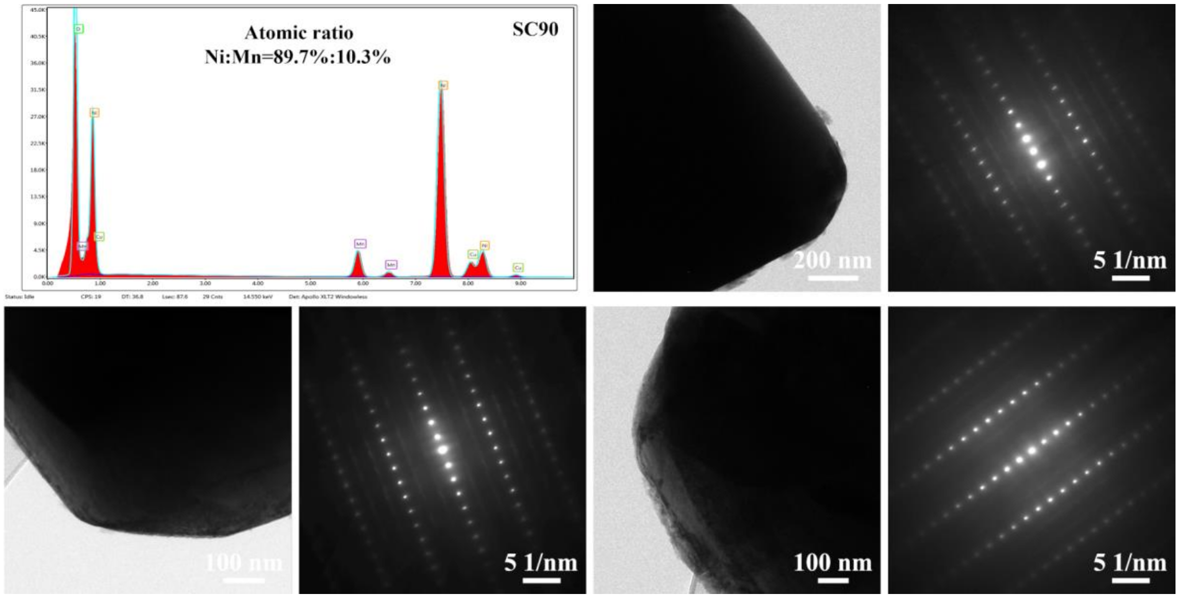


**Supplementary Fig. 13 |** The EDS spectrum and structural characterization of the SC90 sample.

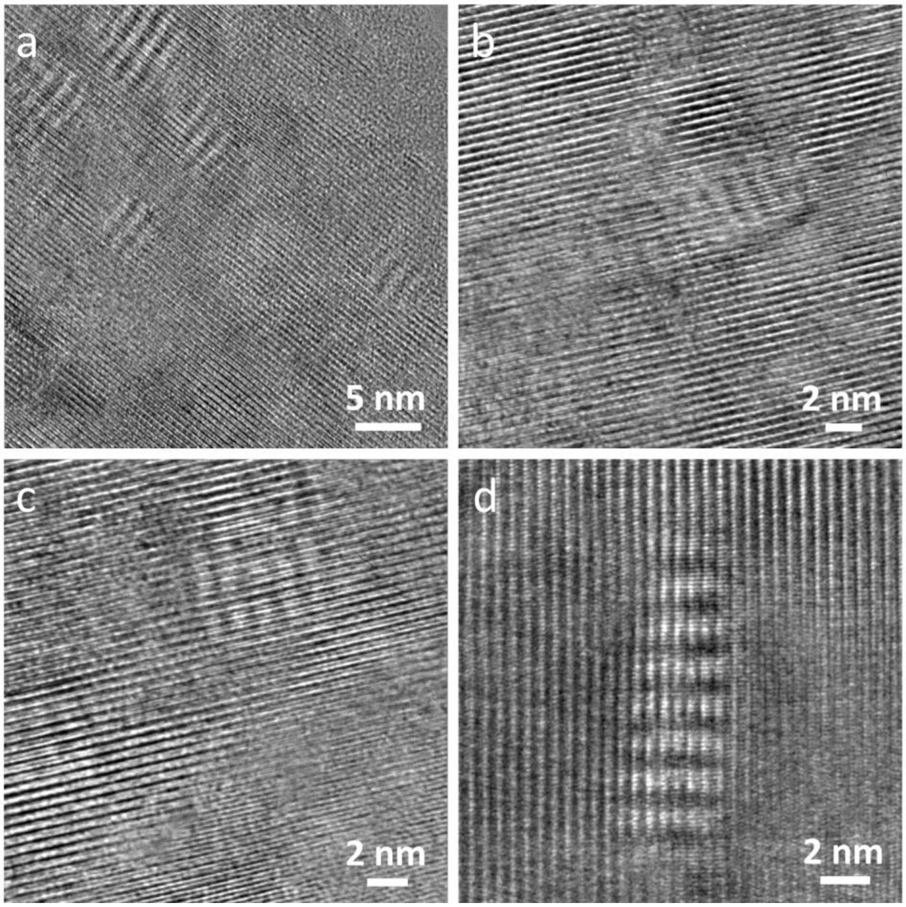


**Supplymentary Fig. 14 | a-d**, The high-magnification TEM images of the SC75 cathode after 100 cycles at 0.5C and 2.8-4.4 V, showing the moire patterns.

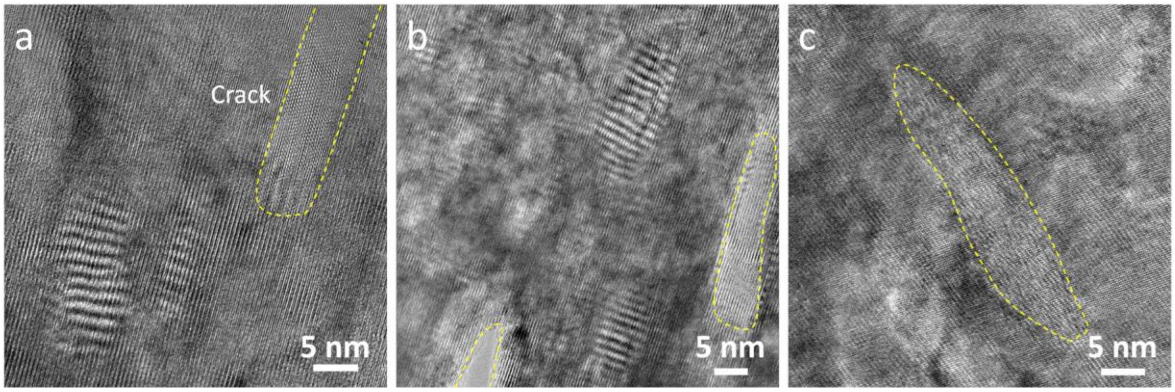


**Supplymentary Fig. 15 | a-c**, The high-magnification TEM images of the SC75 cathode after 100 cycles at 0.5C and 2.8-4.6 V, showing the moire patterns and cracks.
